# In vitro investigation of the impact of pulsatile blood flow on the vascular architecture of decellularized porcine kidneys

**DOI:** 10.1038/s41598-021-95924-5

**Published:** 2021-08-20

**Authors:** Peter R. Corridon

**Affiliations:** 1 Department of Immunology and Physiology, College of Medicine and Health Sciences, Khalifa University of Science and Technology, PO Box 127788, Abu Dhabi, UAE; 2 Wake Forest Institute for Regenerative Medicine, Medical Center Boulevard, Winston-Salem, NC 27157-1083 USA; 3 Healthcare Engineering Innovation Center, Khalifa University of Science and Technology, PO Box 127788, Abu Dhabi, UAE; 4 Center for Biotechnology, Khalifa University of Science and Technology, PO Box 127788, Abu Dhabi, UAE

**Keywords:** Biomedical engineering, Biomaterials, Implants, Tissues, Nephrology, Physiology, Circulation, Kidney

## Abstract

A method was established using a scaffold-bioreactor system to examine the impact pulsatile blood flow has on the decellularized porcine kidney vascular architecture and functionality. These scaffolds were subjected to continuous arterial perfusion of whole blood at normal physiological (650 ml/min and 500 ml/min) and pathophysiological (200 ml/min) rates to examine dynamic changes in venous outflow and micro-/macrovascular structure and patency. Scaffolds subjected to normal arterial perfusion rates observed drops in venous outflow over 24 h. These reductions rose from roughly 40% after 12 h to 60% after 24 h. There were no apparent signs of clotting at the renal artery, renal vein, and ureter. In comparison, venous flow rates decreased by 80% to 100% across the 24 h in acellular scaffolds hypoperfused at a rate of 200 ml/min. These kidneys also appeared intact on the surface after perfusion. However, they presented several arterial, venous, and ureteral clots. Fluoroscopic angiography confirmed substantial alterations to normal arterial branching patterns and patency, as well as parenchymal damage. Scanning electron microscopy revealed that pulsatile blood perfusion significantly disrupted glomerular microarchitecture. This study provides new insight into circumstances that limit scaffold viability and a simplified model to analyze conditions needed to prepare more durable scaffolds for long-term transplantation.

## Introduction

Nearly a billion individuals worldwide are affected by acute and chronic kidney conditions^1^. Presently, there are no cures for either acute kidney injury (AKI) or chronic kidney disease (CKD), and the prevalence of these debilitating diseases is on the rise. The escalating incidences of both conditions produce overwhelming burdens on healthcare systems, and studies have reported high rates of transition from AKI to CKD^2^. These renal complications support progressive and irreversible damage that often leads to end-stage renal disease (ESRD)^3^. This devastating disease progression illustrates a growing public health issue that results in high morbidity and mortality rates^4^, and highlights the need to improve kidney disease management.

Once a renal disease progresses to ESRD, the kidneys can no longer perform their normal functions. At that point, clinical options are limited to renal replacement therapy (RRT), which consists of various treatment modalities that replace the normal filtration, secretion, reabsorption, endocrine, and metabolic functions of the kidney in varied capacities. These modalities are often grouped into two main categories: dialysis and transplantation^5^. While dialysis techniques replace lost filtration capacities, which help remove toxins and regulate blood pressure and pH, transplantation is the ultimate solution to reinstate all innate kidney functions. Unfortunately, the severe global shortage of transplantable kidneys^6^, as well as organ rejection to a lesser extent^7^, limit this ideal option and accentuate the demand for alternative solutions.

Fortunately, recent advances in tissue engineering and regenerative medicine may help address this need. Such advances support the development of bioartificial kidneys, which can potentially increase the number of available transplantable kidneys, reduce transplant rejection rates, and significantly diminish morbidity and mortality in patients with acute and chronic disorders^8^. One viable pathway to developing transplantable devices relies on whole organ decellularization to create natural scaffolds^9^. Researchers use this technique to create scaffolds by isolating the intact extracellular matrix (ECM) from human^10^, canine^11^, ovine^12^, and porcine kidneys^13^. Acellular scaffolds then provide a platform for cell growth, differentiation, and tissue/organ development.

In the last few years, significant efforts have been made to optimize whole kidney decellularization techniques. Studies conducted on porcine kidneys have provided promising results with perfusion decellularization protocols using detergents^13–16^. For instance, it was shown that porcine kidneys decellularized using only sodium dodecyl sulfate (SDS) retained an intact vascular tree and essential ECM architecture, while maintaining vascular patency within the first two hours after transplantation^17^. Similar studies that investigated the vascular patency for longer periods showed that these scaffolds developed thrombosis of the entire vascular tree after implantation. Nevertheless, the vascular architecture appeared intact, and the scaffolds sustained blood pressure in vivo for two weeks^16^. An alternative method that relied on Triton X-100 and SDS successfully removed cellular components and preserved ECM and vascular architectures. This study further provided evidence of the efficient removal of residual DNA from the scaffolds and the protocol has been identified as a reliable method to decellularize whole porcine kidneys^14^. Altogether, these results have provided crucial insight, but there is still a gap in the current understanding of decellularized scaffold vascular integrity, in general, and specifically, in post-transplantation settings^18^. Further studies are needed to characterize the structural and functional capacities of the decellularized vasculature. It is essential to understand how these properties are affected by physiological and pathophysiological conditions and how these conditions influence the clinical applications of decellularized organs.

Therefore, the objective of this study was to establish a method to investigate the integrity of vascular networks in decellularized tissues. A scaffold-bioreactor system was created to subject acellular organs to continuous perfusion of whole blood, at normal and abnormally low rates, to examine dynamic changes in venous outflow and micro-/macrovascular structure and patency. Such studies can help examine this technology's clinical utility and offer a comparative analysis of various disease states that impede transplantation, such as arterial hypoperfusion stemming from renal stenosis.

## Results

### Assessment of whole organ decellularization

**Figure 1:**
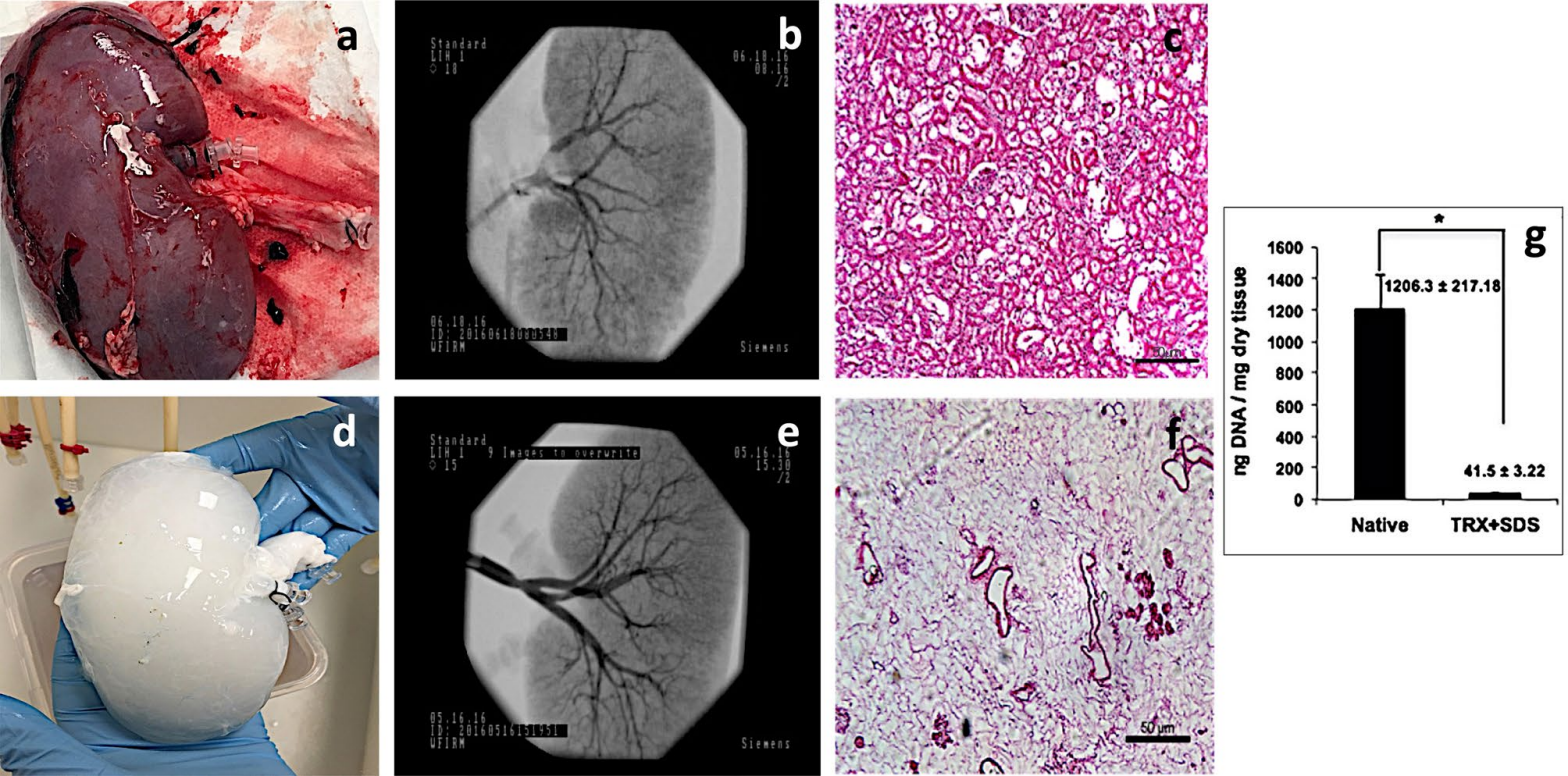
Perfusion decellularization of whole porcine kidneys. A photograph (**a**), angiographic analysis (**b**), and histologic analysis (H&E) (**c**) of the native kidney. A photograph (**d**), angiographic analysis (**e**), and histologic analysis (H&E) (**f**) of the decellularized native kidney. DNA quantification for native and decellularized kidneys (**g**). Images (**c**), (**f**) and (**g**) are reprinted from Acta Biomaterialia, Volume 75, Zambon, J. P.; Ko, I. K.; Abolbashari, M.; Huling, J.; Clouse, C.; Kim, T. H.; Smith, C.; Atala, A.; and Yoo, J. J., Comparative analysis of two porcine kidney decellularization methods for maintenance of functional vascular architectures, pp. 226–234, Copyright (2018), with permission from Elsevier. Scaffolds used for the current study and those used in this referenced study (Zambon et al.) were all decellularized simultaneously and under the same conditions in a high-throughput system and were part of the original evaluation outlined in the referenced study. Scale bars represent 50 μm.

**Figure 2:**
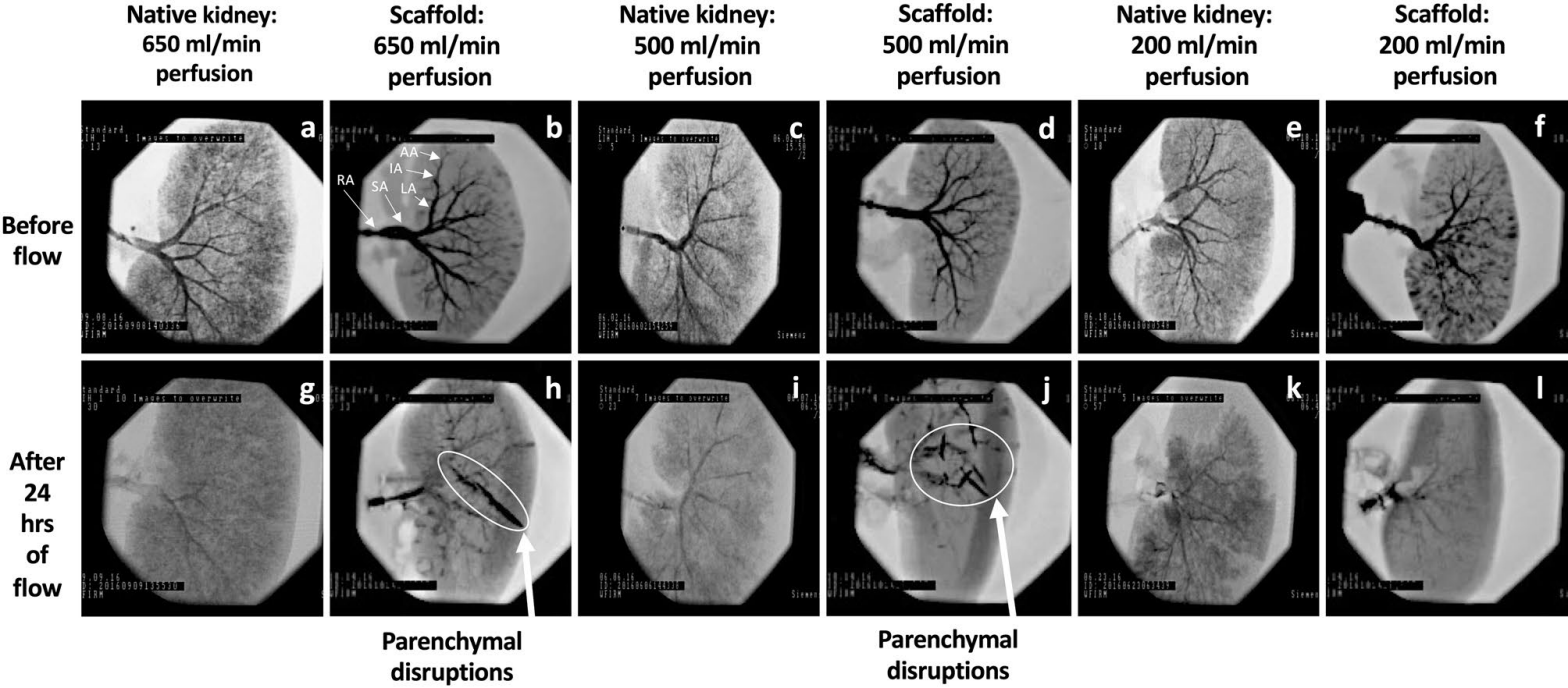
Fluoroscopic angiography shows the impact of the continuous perfusion of unreplenished and unfiltered blood for 24 h on the native and decellularized kidney vasculature. Angiograms taken before the kidneys were perfused with blood display the main renal artery (RA), segmental artery (SA), lobar artery (LA), interlobar artery (IA), and arcuate artery (AA) (**a**) through (**f**). Angiograms taken after 24 h of blood perfusion at rates of 650 ml/min (**g**), 500 ml/min (**i**), and of 200 ml/min (**k**) from native kidneys, and decellularized scaffolds perfused at rates of 650 ml/min (**h**), 500 ml/min (**j**), and 200 ml/min (**l**) all display disruptions to the previously intact vascular network. The arrows in images (**h**) and (**j**) are used to indicate ruptures to the scaffold parenchyma revealed by angiography that were not visible from the surface. The higher flow rates also appeared to generate visible parenchymal damage in the scaffolds.

Perfusion decellularization produced a discernible shift in color from the native (Fig. 1a) to the decellularized (Fig. 1d) organs. Fluoroscopic angiography revealed that the vascular network was preserved after decellularization by comparing the hierarchical branching structures in the native kidneys (Fig. 1b) to those in the acellular organs (Figs. 1e, 2a–c). Histological analysis showed the typical presence of cellular and extracellular matrix components in the native kidney (Fig. 1c). In comparison, the decellularization process facilitated the removal of native cellular and extracellular components, as previously reported by Zambon et al. (Fig. 1f). DNA quantification analysis in that study also presented evidence that perfusion decellularization removed over 95% of the DNA from native kidneys (Fig. 1g).

### Characterization of the scaffold micro-/macrovascular integrity after blood perfusion

Angiograms taken from native and decellularized kidneys before and after perfusion with unreplenished and unfiltered blood for 24 h revealed substantial alterations in the decellularized vascular network (Fig. 2). Before perfusion, the main renal artery and segmental, lobar, interlobar, and arcuate arteries were visible (Fig. 2a–f). However, standard arterial branching patterns were considerably disrupted by the end of perfusion, and at that point, it was difficult to detect and distinguish the various branches of the arterial tree (Fig. 2g–l).

After 24 h of hypothermic blood perfusion, the native and acellular kidneys that were perfused at normal arterial inflow rates were unable to maintain a standard vascular architecture. Furthermore, these changes in vascular patency were accompanied by substantial damage throughout the parenchyma of the decellularized scaffolds (Fig. 2h,j), which were not visible from the surface. The higher flow rates also appeared to generate visible parenchymal damage in the scaffolds, whereas such damage was not observed in the native kidneys (Fig. 2g,i,k) and scaffolds perfused at an abnormally lower perfusion rate of 200 ml/min (Fig. 2l). These vascular networks were also impaired in different ways. Specifically, there were instances when the scaffold integrity was compromised more in the lower pole than in the upper pole and vice versa. Moreover, the acellular kidneys that received 200 ml/min arterial inflow showed far less of an ability to perfuse blood throughout their vascular networks, in a manner similar to what was observed in the native kidneys after perfusion. In these hypoperfused native kidneys and scaffolds, there were more notable physical signs of thrombosis and vascular damage around the medial borders (Fig. 2k,l) that would have resulted in subsequent impairment throughout the vasculature.

**Figure 3:**
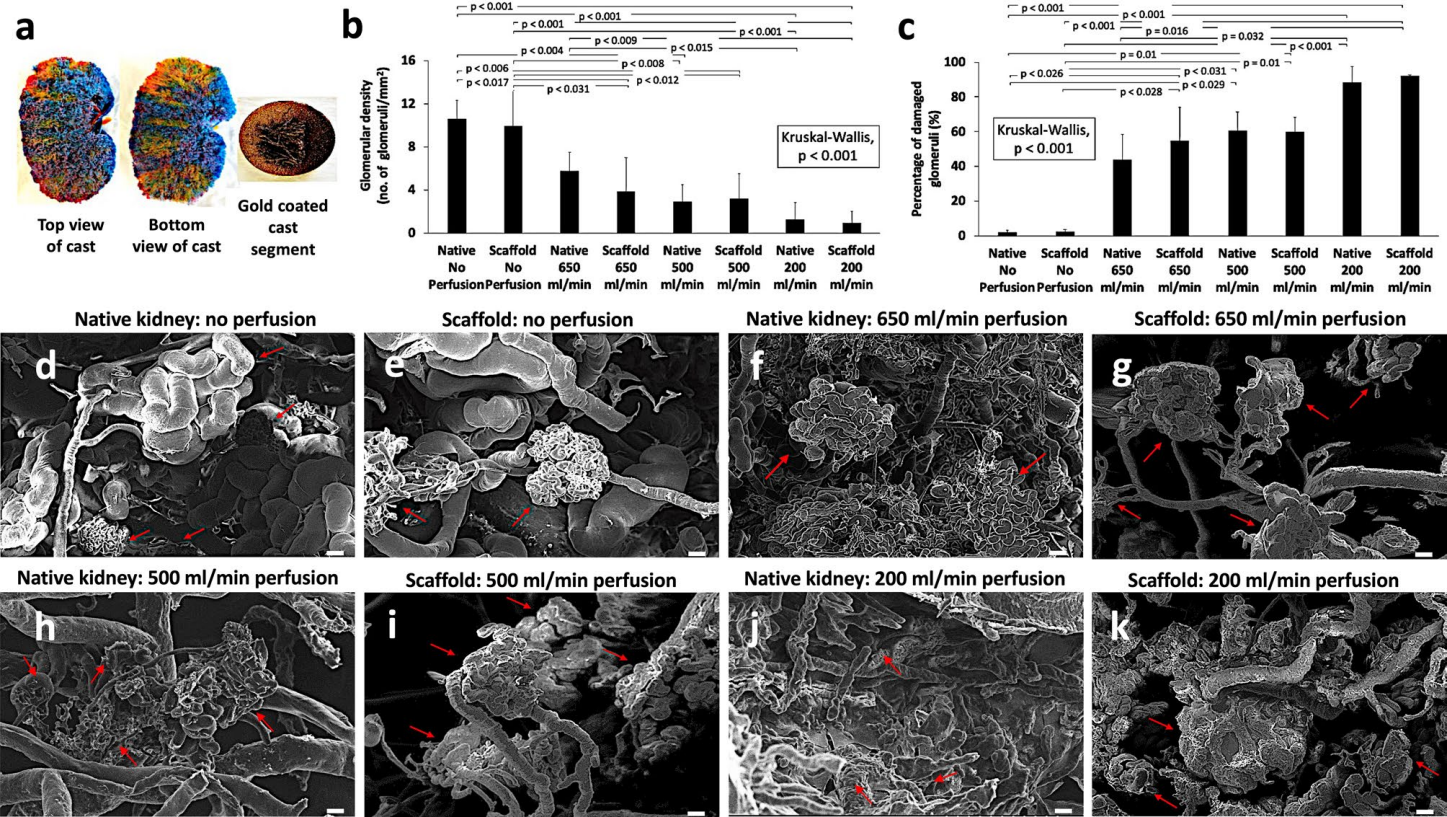
SEM  analysis to evaluate the disruption to glomerular microarchitecture in native kidneys and decellularized scaffolds. Two photographs of a polymethylmethacrylate vascular corrosion cast outing the arterial (red), venous (blue), and ureter/pelvis/calyx (yellow) tracks viewed from the top and bottom of the cast, and an arterial portion of the vascular corrosion cast that was sputter-coated with gold for imaging by the SEM (**a**). Comparison of the percentages of damaged glomeruli recorded in native kidneys and decellularized scaffolds subjected to blood perfusion at the various rates of perfusion (b). Comparison of the glomerular densities recorded in native kidneys and decellularized scaffolds that were subjected to blood perfusion at the given perfusion rates (**c**). The p values are based on the Kruskal–Wallis test (*p* < 0.001), and pairwise group comparisons are illustrated by means of the Dunn’s post hoc test to highlight the pairs that were significantly different. SEM images of a native kidney (**d**) and decellularized scaffold (**e**) that were not subjected to blood perfusion, as well as images taken from native kidneys that was perfused for 24 h at a rate of 650 ml/min (**f**), 500 ml/min (**h**), 200 ml/min (**j**), and from scaffolds also subjected to 24 h of blood perfusion at rates of 650 ml/min (**g**), 500 ml/min (**i**), and 200 ml/min (**k**). Red arrows in images (**d**) through (**k**) identify intact or damaged glomerular structures within the kidneys. Scale bars represent 40 μm.

Scanning electron microscopy (SEM) analysis conducted on portions of the arterial vasculature, which were extracted from the resin corrosion casts and coated with an ultra-thin coating of gold (Fig. 3a), allowed the examination of the ultrastructural architecture in native kidneys and decellularized scaffolds before (Fig. 3d,e) and after (Fig. 3f–k) perfusion-based studies. From a qualitative perspective, the images obtained provided the visualization of the three-dimensional appearance of the glomerular tuft and associated capillaries. In the non-perfused samples, the vascular structural integrity was largely preserved. However, substantial disruptions to these structures resulted from the 24-h perfusion of non-replenished blood irrespective of the perfusion rate, whereby the glomerular tufts and other capillaries appeared constricted, torn, fragmented, and, in many cases, altogether absent. Other alterations in the microvascular structure included a loss of their uniform appearance after perfusion and the widespread development of larger pores across the surfaces.

To gain further insight on these results, a quantitative investigation was also performed. In this investigation, the percentage of damaged glomeruli and changes in the glomerular density were assessed among the native kidneys and decellularized scaffolds perfused with whole blood at rates of 650, 500, and 200 ml/min. The Kruskal–Wallis one-way analysis of variance (ANOVA) provided evidence of a difference (H = 35.294, *p* < 0.001) between the mean percentages of damaged glomeruli of at least one pair of the groups (Fig. 3b).

Moreover, post hoc Dunn’s pairwise tests revealed significant differences between the following pairs: native non-perfused kidneys and native kidneys perfused at 500 ml/min (*p* = 0.029) and 200 ml/min (*p* < 0.001); native non-perfused kidneys and decellularized scaffolds perfused at 650 ml/min (*p* = 0.026), 500 ml/min (*p* = 0.01), and 200 ml/min (*p* < 0.001); non-perfused decellularized scaffolds and native kidneys perfused at 500 ml/min (*p* = 0.025) and 200 ml/min (*p* < 0.001); non-perfused decellularized scaffolds and decellularized scaffolds perfused at 650 ml/min (*p* = 0.028), 500 ml/min (*p* = 0.01), and 200 ml/min (*p* < 0.001); native kidneys perfused at 650 ml/min and native kidneys perfused at 200 ml/min (*p* = 0.032); and native kidneys perfused at 650 ml/min and decellularized scaffolds perfused at 200 ml/min (*p* = 0.016).

This non-parametric ANOVA test also provided evidence of a difference (H = 32.878, *p* < 0.001) between the glomerular densities of at least one pair of the groups (Fig. 3c). Likewise, the post hoc Dunn’s pairwise tests in this case revealed significant differences between the following pairs: native non-perfused kidneys and native kidneys perfused at 500 ml/min (*p* = 0.004) and 200 ml/min (*p* < 0.001); native non-perfused kidneys and decellularized scaffolds perfused at 650 ml/min (*p* = 0.017), 500 ml/min (*p* = 0.006), and 200 ml/min (*p* < 0.001); non-perfused decellularized scaffolds and native kidneys perfused at 500 ml/min (*p* = 0.008) and 200 ml/min (*p* < 0.001); non-perfused decellularized scaffolds and decellularized scaffolds perfused at 650 ml/min (*p* = 0.031), 500 ml/min (*p* = 0.012), and 200 ml/min (*p* < 0.001); native kidneys perfused at 650 ml/min and native kidneys perfused at 200 ml/min (*p* = 0.009); and native kidneys perfused at 650 ml/min and decellularized scaffolds perfused at 200 ml/min (*p* = 0.015).

### Alterations in scaffold venous outflow

**Figure 4:**
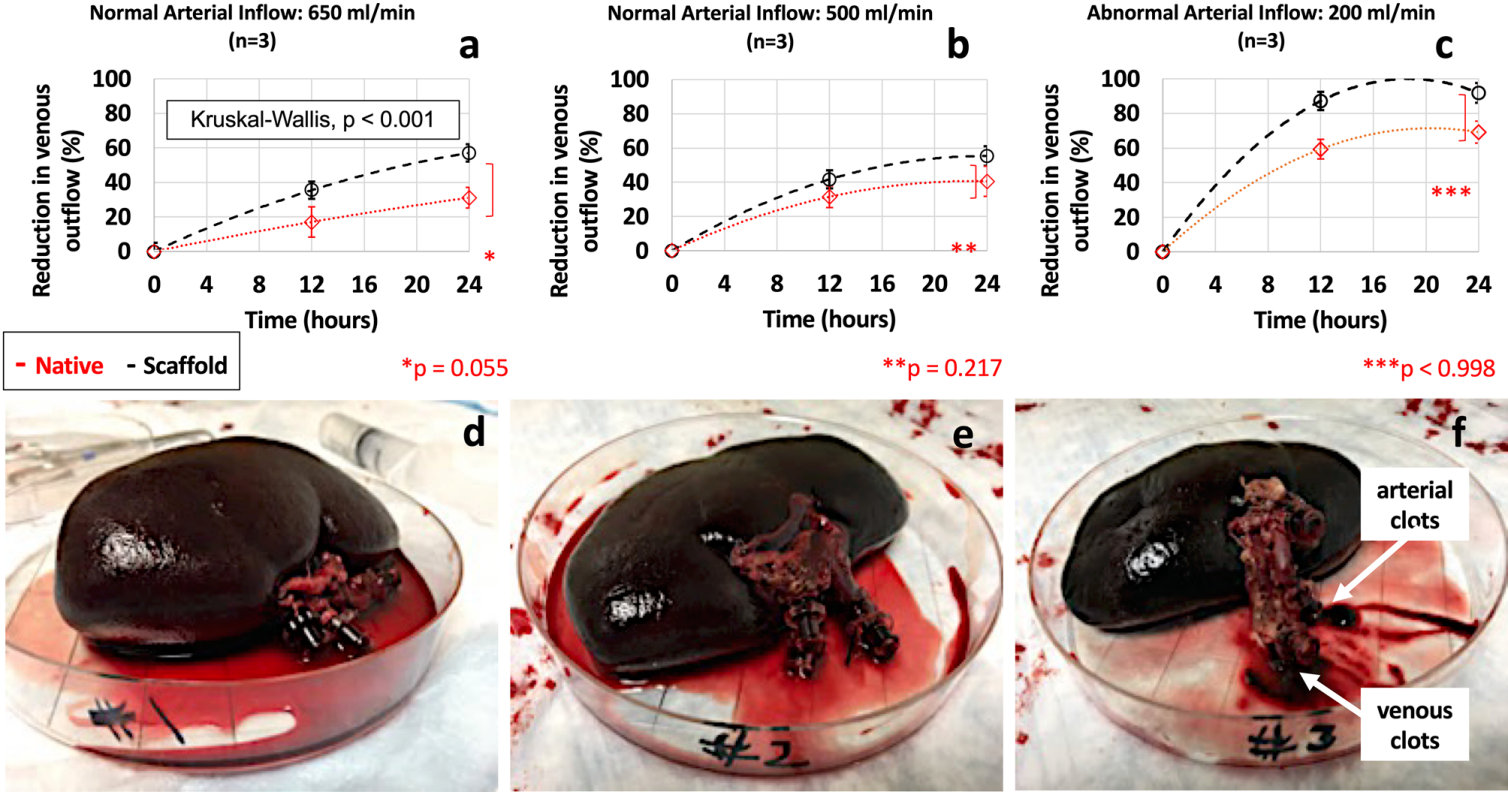
Plots of the time-dependent reductions in venous blood outflow and photographs of kidneys after perfusion. Time-dependent curves display the drops in the venous outflow from decellularized scaffolds subjected to 24 h of blood perfusion at rates of 650 ml/min (**a**), 500 ml/min (**b**), and 200 ml/min (**c**). Data is also presented in images (**a**), (**b**) and (**c**) from control groups of native kidneys perfused with blood for 24 h at 650 ml/min, 500 ml/min and 200 ml/min respectively. Photographs of decellularized scaffolds that were subjected to perfusion at rates of 650 ml/min (**d**), 500 ml/min (**e**), and 200 ml/min (**f**) were taken at the 24-h mark once the perfusion studies were completed. Multiple arterial and venous thrombi were present in scaffolds perfused with blood at a rate of 200 ml/min. The p values presented are based on the Kruskal–Wallis test (*p* < 0.001), and pairwise group comparisons are illustrated by means of Dunn’s post-test, which examined the differences between the control group (native kidneys perfused at 650 ml/min) and decellularized scaffolds perfused at 650 ml/min (*), 500 ml/min (**), and 200 ml/min (***) after 24 h of perfusion.

During the blood perfusion studies, values of the venous output were recorded from native and decellularized kidneys at the start of perfusion (0-h mark), and after 12 h and 24 h. These organs were continuously perfused with unreplenished blood (Fig. 4a–c). Control groups of native kidneys and their associated treatment groups of decellularized scaffolds were perfused with whole blood at 650 ml/min, 500 ml/min, and 200 ml/min. At the beginning of each perfusion study, a steady flow of blood emerged from both the renal vein and ureter. However, as perfusion continued, there were significant reductions in blood outflow throughout the test period.

The native and decellularized kidney samples that were subjected to arterial perfusion rates of 650 ml/min (Fig. 4a) and 500 ml/min (Fig. 4b), had far lower reductions in venous outflow when compared to the native and decellularized organs that received an abnormally low arterial perfusion rate of 200 ml/min (Fig. 4c). Specifically, with an arterial perfusion rate of 650 ml/min, gradual drops in the venous outflow from the native kidneys were observed across the 24 h, outlining that the reductions in venous output rose from 17.075% after 12 h to 31.133% after 24 h. Similarly, native kidneys perfused at 500 ml/min had reductions in venous output, 31.515% after 12 h and 40.570% after 24 h.

In comparison, greater reductions in venous output were recorded at the 12-h, 59.346%, and 24-h, 69.174%, timepoints when native kidneys were perfused at 200 ml/min. Venous outflow data collected from decellularized kidneys subjected to the same experimental conditions showed more substantial reductions in the venous output at all perfusion rates. Namely, the venous outflow dropped by 35.471% after 12 h and 57.077% after 24 h of perfusion at 650 ml/min, and by 41.702% after 12 h and 55.317% after 24 h of perfusion at 500 ml/min. However, the most considerable reductions in venous output observed were recorded from scaffolds perfused at 200 ml/min and were 87.185% after 12 h and 91.850% after 24 h.

At the 24-hour mark, both the native and decellularized kidneys perfused at normal arterial infusion rates still perfused the recirculated and unreplenished blood. Moreover, at the end of the test period, these kidneys appeared intact macroscopically. There were no apparent signs of clotting in samples perfused at 650 ml/min (Fig. 4d) and 500 ml/min (Fig. 4e). However, the kidneys subjected to a far lower perfusion rate of 200 ml/min produced much sharper reductions in the venous outflow. These organs were poorly perfused across the 24 h. Similarly, these hypoperfused kidneys also appeared macroscopically intact at the end of the study but presented several thrombi (Fig. 4f), which drained out of artery, vein, and ureter after disconnecting the organs from the arterial line in the bioreactor system at the end of perfusion.

The Kruskal–Wallis one-way ANOVA again revealed a significant difference (H = 22.229, *p* < 0.001) between the mean reductions in the venous output from native kidneys and decellularized scaffolds perfused at all the given rates (Fig. 4). The Dunn’s test provided evidence that the following pairwise differences in the venous outputs were not significant: native kidneys and decellularized scaffolds perfused at 650 ml/min (*p* = 0.055), 500 ml/min (*p* = 0.217), and 200 ml/min (*p* = 0.998). Whereas the Wilcoxon signed-rank sum test indicated that the reductions in venous output after 12 h (*p* = 0.027) and 24 h (*p* = 0.028) were statistically significant from the venous flow at the beginning of the perfusion studies for samples perfused at 650 ml/min. Likewise, this pairwise test also indicated that the reductions in venous output that occurred at 500 ml/min after 12 h (*p* = 0.028) and 24 h (*p* = 0.028), and at 200 ml/min after 12 h (*p* = 0.026) and 24 h (*p* = 0.026) were statistically significant from the respective venous flow at the beginning of the perfusion studies.

## Discussion

In this study, a method to investigate the integrity of vascular networks in decellularized tissues was developed using a scaffold-bioreactor system. This technique helped determine how pulsatile blood flow impacted the decellularized kidney vascular structure and function. The results showed that acellular scaffolds could better withstand perfusion of unreplenished and recirculated blood at physiologically normal rates of either 650 ml/min or 500 ml/min than at a pathological rate of 200 ml/min. Regardless of the flow rate, substantial disruptions to vascular patency and integrity, as well as venous output, occurred over 24 h.

Few studies have evaluated the integrity and function of the decellularized vasculature in whole pig kidneys under physiological conditions. The majority of these studies have primarily focused on demonstrating the preservation of vascular structure and patency after decellularization and post-implantation. Thus, further research is needed to improve our current understanding. Zambon et al.^19^ found that the sequential combination of detergents efficiently removed cellular components and produced whole decellularized organs with intact vascular architectures. In that study, the authors outlined that 72 h of PBS perfusion removed detergent residuals and cell components, and this protocol also supported the recellularization of the porcine kidneys with endothelial cells. This is an important feature of the protocol as the inefficient removal of SDS from the scaffolds could prolong the decellularization process and damage fibers within the ECM, as well as reduce the effectiveness of the recellularization process^20^. Additionally, the incomplete removal of scaffold remnants, which include cellular and tissue debris generated from the decellularization process, may influence unwanted biological or immunological responses after transplantation^21^. As a result, it was crucial to establish a protocol that prevents such outcomes. Overall, these results provide a valuable pre-test characterization of the decellularization protocol's effectiveness and support its use in these studies.

To the author’s best knowledge, there have not been any reports measuring whole acellular kidney hemodynamic responses at physiologically relevant states to this date. The investigations presented in this manuscript were aimed at providing insight into circumstances that limit scaffold viability. Therefore, it was hypothesized that a method could be established to investigate the integrity of decellularized vascular networks by exposing these organs and tissues to various states that mimic physiological and pathophysiological settings and examine their performance and current suitability for transplantation. Renal blood flow is roughly 20% to 25% of the cardiac output, ranging from 1.0 L/min to 1.2 L/min^22^. The arterial inflow rates of 500 ml/min and 650 ml/min used in this study correspond to the amount of blood each kidney would receive during resting conditions.

Under normal conditions, the kidneys autoregulate renal blood flow to ensure that pressure elevations are not transmitted to glomeruli and capillaries^23^. However, depending on the level of the systolic pressure, impairments to the renal autoregulatory system support capillary hypotension or hypertension^24^. These abnormal states can be compared to that of the decellularized kidney and the native kidney within in vitro settings, which are incapable of autoregulation. It is thus conceivable that the loss of such function would have exposed the decellularized capillaries and glomeruli to damage that stemmed from elevated pressure levels during the perfusion studies conducted at 650 ml/min and 500 ml/min.

By lowering the inflow rate to 200 ml/min, renal blood pressure was substantially reduced to approximately 25 mmHg. This hypoperfused state helped mimic aspects of arterial stenosis, which is a well-recognized process that compromises kidney transplantation^25^. The model did not fully mimic all aspects of transplant renal artery stenosis observed in vivo since the scaffolds could not inherently generate compensatory increases in pressure. However, recirculating unreplenished blood provided an additional means to produce vascular occlusions stemming from coagulation, which would have increased blood viscosity^26^, and supported the onset of renal ischemia^27^. These events would have accompanied red blood cell aggregation and thrombosis^28^, and may explain the multiple thrombi that drained out of the artery, vein, and ureter at the end of the study, as well as the damage observed at the medial borders of native and decellularized kidneys perfused at 200 ml/min.

Again, as the blood was not replenished, the various blood cells, particularly the red blood cells, were continuously exposed to low glucose environments that would have limited glycolysis, which is their sole energy source^29^. These conditions would have supported hemolysis^30^, as well as eryptosis and aggregation^31^, further increasing blood viscosity with time. Such compounded increases in blood viscosity could have, in turn, further supported ischemia and occlusions throughout the kidneys highlighted by the disruptions to the vascular network detected by angiography. Similarly, erythrocytes, leukocyte and platelet activities, which rely on mitochondrial machinery to meet normal energetic demands, would have also been impacted by this deficiency in nutrients. Mitochondrial dysfunction that would have resulted from hypoxia could have facilitated the release of reactive oxygen species resulting in potential damage to proteins and lipids^32^, and further the degradation of the whole organs. Additionally, perfusing the scaffold with whole blood would have given platelets direct access to collagen and enabled their adhesion and activation for thrombus formation^32^.

Fundamental fluid dynamics concepts may also be applied to interpret these results. The steady flow of blood that emerged from the renal vein at the beginning of each experiment signified that each arterial infusion flow rate supported whole organ perfusion. As a result, it was possible to utilize the arterial tree within the scaffold to deliver blood throughout the organ. Nevertheless, as perfusion continued in all cases, there were significant reductions to renal vein blood flow, even though arterial renal blood flow was kept constant. This may suggest that more blood could have been stored in the kidney over time, resulting in stagnation and swelling, which could explain the substantial disruptions to the scaffold parenchyma observed in only decellularized kidneys. In contrast, the internal structures of native kidneys would have been better supported by the innate vascular walls and provided more resistance to such structural deformation from blood perfusion.

According to Hagen-Poiseuille’s law, renal blood viscosity is a critical parameter to support blood flow and could also explain the observed modifications in vascular structure. This law, which states, $Q = \frac{{\pi r^{4} \Delta P}}{8l\eta }$, describes how blood flow ($Q$) can be modeled as a function of blood pressure ($\Delta P$), viscosity ($\eta$), vessel length ($l$), and radius ($r$). From this law, hypothermic perfusion could have been another consequence of recirculating unreplenished and unfiltered blood that would have also led to increases in blood viscosity, and ultimately hampered blood flow regionally and throughout denervated organs now devoid of their innate autoregulative capacities.

Thus, it is feasible that thrombi formation would have occurred throughout the vasculature and impeded flow at the capillary network level, causing substantial glomerular damage observed in the SEM analyses^33^. Moreover, the higher and sustained perfusion rates of 650 ml/min and 500 ml/min would have supported the development of high hydrostatic pressures capable of damaging elastin and collagen fibers to adversely alter ECM physical properties within the scaffolds^34^. Such damage would have ultimately led to a decline in mechanical strength and elastic function^35^. Similarly, the continuous recirculation of the viscous unfiltered and unreplenished blood could have also supported further degradation of sensitive ECM structures, attenuated the scaffolds' mechanical integrity, and explained the severe micro-/macrovascular injury observed. Even though the native kidneys succumbed to the some of the abovementioned effects of the continuous perfusion of such blood, they still possessed sufficient structural barriers that prevented comparable damage to the parenchyma regardless of the perfusion condition.

The current study has some limitations, and further investigations are required to address the small sample size, the nature of the blood used to perfuse the scaffolds, and the length of perfusion investigated. Nevertheless, this investigation showed that kidney scaffolds perfused at physiologically normal rates were still capable of circulating blood of this nature at the end of the study. The higher flow rates may have been sufficient to clear enough cells and tissue debris to maintain higher venous outflow levels by the 24-h mark, comparable to that of the native kidney. This is an important observation, as it displays scaffold durability under harsh conditions, and thus, it is possible to imagine that perfusing the scaffold with filtered and replenished blood may improve functionality.

Overall, it should also be noted that the perfusion process damages the internal structures of both native and decellularized organs. While a significant difference was observed between perfused and non-perfused native kidneys, no significant difference was detected between perfused native and decellularized organs perfused at the same rate. The kidney's functional structure and complexity constitute a great challenge to developing clinical products^36^. Understanding the limitations of the renal decellularized architecture in harsh and detrimental environments will provide insight into the deformation dynamics and biomechanical improvements needed to improve scaffold quality. From a translational perspective, decellularization technologies hold great promise for the bioartificial tissue/organ industry. Therefore, this in vitro approach can be extended for use with other whole organ systems and tissue sections to support their clinical utility.

## Conclusion

A method was established using a scaffold-bioreactor system that examined the impact of pulsatile blood flow on the decellularized vasculature. The decellularized samples and the native samples exhibited the same amount of damage when the same flow rate was used, and the only difference between the groups was the venous output. This scaffold-bioreactor system provides a means to investigate the time-based alterations in decellularized renal vascular integrity and functionality. Its use can be simultaneously extended to other platforms to identify ways to make any decellularized architecture less susceptible to degradation and more viable for long-term transplantation.

## Materials and methods

### Porcine kidney perfusion decellularization and sterilization

Adult Yorkshire pigs were euthanized under the guidelines provided by the Institutional Animal Care and Use Committee (IACUC) at the School of Medicine, Wake Forest University. All experimental protocols complied with all relevant ethical guidelines and regulations provided and approved by this institutional review board, as well as the review board, Animal Research Oversight Committee (AROC), at Khalifa University of Science and Technology to ensure that animals were treated ethically and humanely. Moreover, all methods were performed in accordance with the ARRIVE guidelines. Whole porcine kidneys, with renal arteries, veins, and ureters, were decellularized and sterilized using previously established methods^19^. Decellularization occurred in a non-sterilized environment. A sequential combination of detergents (Triton X-100 and SDS) and phosphate-buffered saline (PBS) was slowly infused into cannulated renal arteries, each at a rate of 5 ml/min. First, 1% Triton X-100, which was dissolved in deionized water, was perfused through the renal artery for 36 h. Second, 0.5% SDS in PBS was infused for an additional 36 h. Last, the kidneys were perfused with PBS for 72 h to remove residual traces of detergents and cellular components. After decellularization, the scaffolds were submerged in PBS and then sterilized with 10.0 kGy gamma irradiation^13^.

### Fluoroscopic angiography

Whole native and decellularized kidneys were first flushed with PBS (approximately 100 ml) via the renal artery. Iothalamate meglumine contrast agent (60% Angio-Conray, Mallinckrodt Inc., St Louis, MO, USA) was then infused into the artery. Once a sturdy flow of contrast exited the renal vein, the renal vein, renal artery, and ureter were occluded to prevent contrast agent from leaking out the organ. Angiograms were conducted on native and decellularized kidneys before and after the scaffolds were perfused with blood, at ambient temperature in a sterilized suite, using a Siemens C-arm Fluoroscope (Siemens AG, Munich, Germany).

### Histological assessment and DNA quantification

To evaluate the perfusion decellularization protocol's effectiveness, sections from native and decellularized kidneys were incubated in neutral buffered formalin (10%, 100 ml solution) at room temperature for 24 h and then embedded into paraffin blocks. The blocks were then sectioned with a microtome to produce 4–5 µm thick samples, which were then mounted on slides and counterstained with hematoxylin and eosin (H&E). Brightfield images were collected and processed from the slides using a Leica DM4000B automated upright microscope (Leica Microsystems, Inc., Buffalo Grove, IL, USA).

DNA concentrations from native and decellularized kidney sections were extracted with a Qiagen DNeasy Kit (Qiagen Inc., Valencia, CA, USA) by first mincing the tissue sections and storing them in a − 80 °C chamber overnight. The samples were then lyophilized, and the DNA concentrations in native kidneys and residual DNA contents in the scaffolds were estimated as a ratio of ng DNA per mg dry tissue. The DNA concentrations within the extracts were quantified using a Quant-iT PicoGreen dsDNA assay kit (Invitrogen Corp., Carlsbad, CA, USA) and a SpectraMax M Series Multi-Mode Microplate Reader (Molecular Devices Inc., Sunnyvale, CA, USA).

### Blood perfusion studies

The following bioreactor components were sterilized using a 60Co Gamma Ray Irradiator: suction pump heads (Ismatec, Cole-Palmer, Wertheim, Germany), standard pump tubing female and male luer × 1/8ʺ hose barb adapters, barbed fittings; reducing connectors, three-way stop cocks, and Kynar adapters (Cole-Palmer, Vernon Hills, IL, USA). The bioreactor tubing, chambers, and 2000 ml round wide mouth media storage bottles with screw cap assemblies (Sigma-Aldrich, St. Louis, MO, USA) were all sterilized using an autoclave.

**Figure 5:**
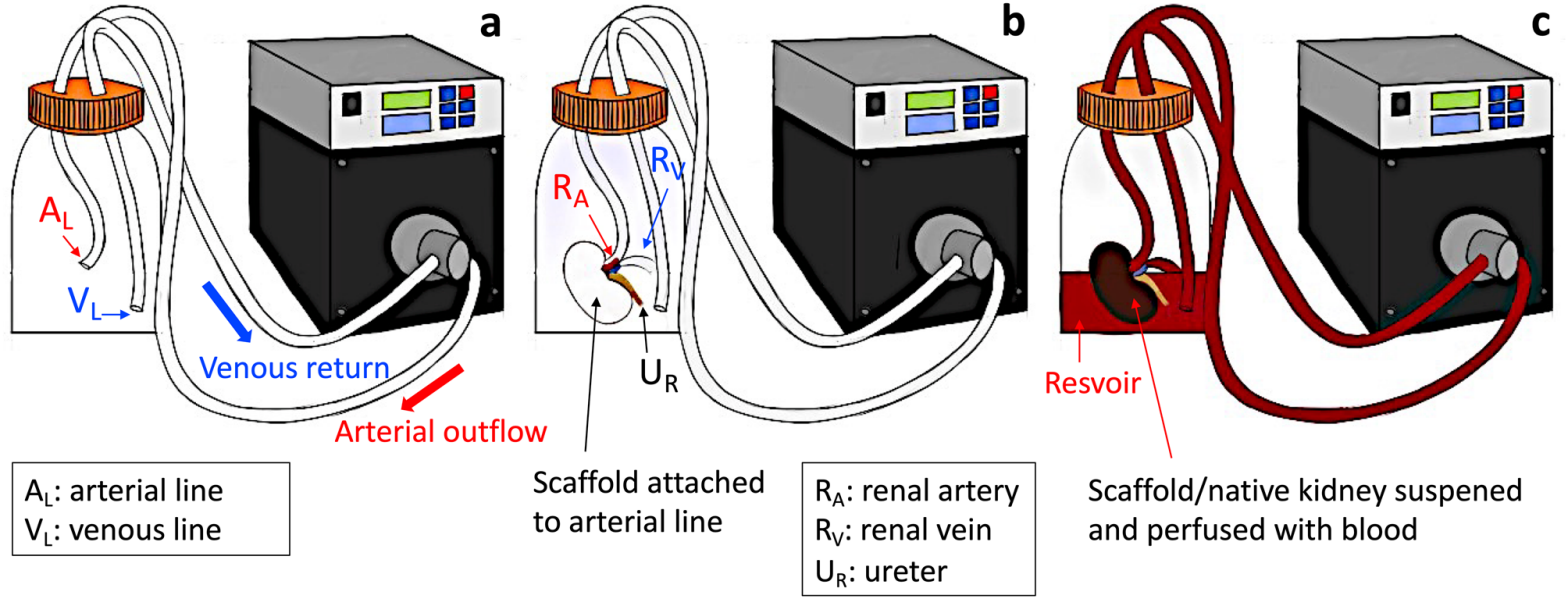
A schematic of the bioreactor model used to perfuse native kidneys and decellularized scaffolds with whole blood. This image illustrates the bioreactor assembly with the arterial (A_L_) that receives blood from the arterial outflow and venous (V_L_) lines that returns blood dispensed from the kidneys for recirculation via the venous return segment (**a**). A decellularized kidney is suspended in the bioreactor, before blood perfusion, by attaching the cannulated renal artery (R_A_) to the arterial line, while the renal vein (R_V_), venous line, and ureter (U_R_) were all left open to support outflow from the kidney sample and venous return to the pump (**b**). A native/decellularized kidney is attached to the arterial line within the assembly and suspended in a reservoir of approximately 400–500 ml of blood to facilitate perfusion at various rates.

After sterilization, the bioreactor systems were assembled within a biosafety cabinet and prepared for perfusion studies, as shown in Fig. 5. The chamber was assembled so that the two outer blood flow lines were attached on either side of a suction pump head. This conformation facilitated arterial outflow from the Ismatec MCP-Z Process or MCP-Z Standard programmable dispensing pump (Cole-Palmer, Vernon Hills, IL, USA) into the chamber’s arterial line and venous return to the pump via the venous line. The renal artery of each scaffold was attached to the arterial line within the chamber, and the scaffold was suspended in a reservoir of approximately 400–500 ml of heparinized pig whole blood in the bioreactor chamber (BioIVT, Westbury, NY, USA). The renal vein cannula was left open to support venous outflow  from the scaffold and back into the reservoir, and the venous line was freely suspended in the reservoir to support its recirculation through the dispensing pumps. The assembled bioreactor systems were then placed in cell culture incubators, and perfusion studies were conducted. The scaffolds were separated into three groups according to the blood perfusion rate: kidneys in group 1 (n = 3) were perfused at a rate of 650 ml/min; kidneys in group 2 (n = 3) were perfused at a rate of 500 ml/min, and kidneys in group 3 (n = 3) were perfused at a rate of 200 ml/min.  Three control groups were used, which consisted of native kidneys perfused at 650 ml/min, group 4 (n = 3), 500 ml/min, group 5 (n = 3), and 200 ml/min, group 6 (n = 3).

The dispensing pumps were programmed to generate ml/rev pulsatile perfusion of the unreplenished and unfiltered blood with 1-s-long fluctuations. The systems were calibrated to determine the rotational speeds (measured in RPM) that produced the desired volume flow rates (measured in ml/min). The associated mean perfusion pressures were 97.21 mmHg, 87.56 mmHg, and 25.46 mmHg for the respective flow rates of 650 ml/min, 500 ml/min, and 200 ml/min. The perfusion pressures were recorded with a digital differential pressure manometer (Dwyer Instruments, Michigan City, IN, USA) by connecting the arterial line to a three-way stop cock that directed flow into the pressure manometer. At each measurement time point, the venous output was sampled to measure the blood volume that exited from the renal vein within a minute. At the 24-h time point, perfusion was ceased, and the scaffolds were removed from the chambers and placed in 60 × 15 mm sterilized polystyrene Petri dishes (Sigma-Aldrich, St. Louis, MO, USA) for fluoroscopic angiography.

### Scanning electron microscopy

To perform additional ultrastructural analyses, multi-colored specimens of native and decellularized kidneys were created using Batson’s #17 Anatomical Corrosion Kit (Polysciences, Inc., Warrington, PA, USA) 18. The polymethylmethacrylate vascular corrosion casts were created by first combining 10 ml of the catalyst (a mixture of acetone and benzoyl peroxide and dibutyl phthalate) and 6 drops of the promoter (dibutyl phthalate + n, n-dimethyl-4-toluidine) with 50 ml of the methyl methacrylate monomer to prepare the injectate polymer solution. The mixture was then subdivided into three portions for infusion into the renal artery (20 ml), vein (20 ml), and ureter (10 ml). The arterial, venous, and ureteral portions were mixed with red, blue, and yellow pigments (10% final concentration for each pigment) to prepare the three final injectates. The three injectates were infused at a 1 ml/min via three separate programmable syringes infusion pumps (New Era Pump Systems, Inc., Farmingdale, NY, USA). After these infusions, native and decellularized kidney samples were placed in a chilled water bath for 3 h to facilitate polymerization. The scaffolds were then submerged overnight in a 20% sodium hydroxide (NaOH) solution at 60 °C.

The next day, the kidney vascular casts were removed from the NaOH solution and air-dried overnight. Extracted portions of the casts were sputter-coated with gold using a Hummer 6.2 Sputter Coater (Anatech USA, Union City, CA, USA). SEM analysis was then conducted on the vascular casts using a Hitachi S-570 (SEM) (Hitachi Hi-Tech, Schaumburg, IL, USA). Micrographs were collected at 200× and 250× magnification with an accelerated voltage of 15 kV. The level of disruption to glomerular microarchitecture was assessed by estimating the percentage of glomeruli with preserved ultrastructure within 3 randomly chosen adjacent fields obtained from 2 cortical sections of each native/decellularized kidney within a given group. Similarly, the glomerular density was determined from the number of glomeruli within the adjacent fields (normal and damaged) divided by the cortical area.

### Statistical analysis

Non-parametric statistics were applied to analyze the data using SPSS (IBM Corp, Armonk, NY, USA). The Kruskal–Wallis one-way analysis of variance (ANOVA) with the post hoc Dunn’s test was used to compare the percentages of damaged glomeruli, glomerular densities, and drops in venous output among the native kidneys and decellularized scaffolds that were perfused with whole blood at rates of 650, 500, and 200 ml/min. The Wilcoxon signed-rank test was used to determine whether the reductions in venous outflow observed at 12-h and 24-h time points were significant. All variables are expressed as mean ± standard deviation, and for all evaluations, a *p* value of less than 0.05 was considered statistically significant.

## Data Availability

The datasets generated during and/or analyzed during the current study are available from the corresponding author on reasonable requests.
